# Bibliographical Notices

**Published:** 1841-01-01

**Authors:** 


### Periscope;

OR,

### CIRCUMSPECTIVE REVIEW.

"Ore trahit quodcunque potest, atque addit acervo."

### Notice of "The Invalids' Guide to Madeira. By. *W. W. Cooper.*"

There are few situations more embarrassing to a medical man, than when he feels himself under the necessity of recommending his patients to resort to a distant climate for the restoration of health. For no one would hastily, or without due consideration, incur the responsibility of advising a person exhausted by disease and suffering to encounter the miseries of a sea voyage, and the discomforts necessarily attendant on a residence in a strange land—or, on the other hand, of recommending such an expedient where change of air was not essential to the safety of his patient.

Experience, however, has taught us that change of climate is the most powerful means we possess of arresting the progress of pulmonary, and other fatal maladies; and that where a timely removal is effected, the most beneficial results may, in a great majority of instances, be expected to follow. It becomes then an important part of a medical man's duty to be able to detect the primary symptoms of consumption, and other structural diseases, which are usually insidious in their approach; and, under ordinary circumstances, fatal in their termination. In pulmonary affections we fortunately possess an auxiliary in the stethoscope, by which we can detect organic changes in the chest, generally before the patient is aware of their existence. It is in these incipient stages of disease that the sanatory and restorative influence of change of climate is so particularly available: and if then had recourse to, no doubt the number of victims to consumption and other fatal maladies would be materially diminished.

Madeira has long and deservedly enjoyed a high reputation for the salubrity of its climate and the equality of its temperature: and in those morbid and cachectic conditions of the system to which we have adverted, a temporary residence there may be expected to produce the greatest benefit.

The author of the little work which has called forth these remarks, takes a similar view of the subject, and strongly enforces the necessity, as well as the great utility, of a timely retreat to this island.

"I would have all beware of delay where there is the slightest tendency to consumption. The approach of that disease is so insidious, its first symptoms so slight, that when judging from appearances alone, it may be said that the individual is only *threatened* with it, at that very time may the rudiments of disease not only be established, but even considerable progress made. When the experienced stethoscopist detects the least symptom of a tubercular condition of the lungs, not a moment should be lost. The position of the individual is fraught with danger, but a timely retreat to such a climate as Madeira, may then be confidently looked forward to as a means of checking the impending danger, and cutting short the disease before it is too late."

The work is written in a very pleasing and agreeable style, and contains much useful information respecting the accommodation, expenses, the customs, and amusements, &c. of the inhabitants; which will be read with great interest by all who visit Madeira either in search of health, business, or pleasure. The medical part of the book is somewhat meagre, and contains but little either of interest or of novelty. But the object of the author has evidently been to furnish (what has long been wanted) a cheap, useful, and entertaining guide to Madeira; rather than to impart information to medical men: in this he has completely succeeded, and we cordially recommend his vade mecum to the notice of our readers.

---

On Diseases of the Hip-Joint; with Observations on Affections of the Joints in the Puerperal State. With Plates. By *W. Coulson*, Surgeon to the Magdalen Hospital, Consulting Surgeon to the City of London Lying-in-Hospital, Fellow of the Royal Medico-Chirurgical Society of London, Corresponding Member of the Medico-Chirurgical Society of Berlin, &c. &c. London, Longman and Co.

In his Advertisement Mr. Coulson informs us that:—"The present edition of this work will be found to differ very materially in form from the first, and to have received considerable additions. The alteration in the arrangement I have adopted in deference to suggestions which appeared to me judicious, and with a view to make the work more readily available in practice. I have treated more fully than in the former edition of the affections with which the diseases of the hip may be confounded; and I have added a chapter (which may be of value for the cases it contains) on the puerperal affections of the joints, to which, according to M. Dugés, the hip is more than any other liable.

In my investigation of the diseases of the hip-joint, I regret that I have not yet been able to classify the inflammatory affections according to the structure primarily affected. During the last sixteen or seventeen years, I have not omitted any opportunity which has occurred to me of examining after death the morbid changes in the part. In all the cases I have examined, where the disease could be said to have been in its earliest stage,—the synovial membrane was inflamed, whilst the cartilages and bone were but very slightly affected at the attachment of the ligamentum teres to the head of the femur. In the other cases, all the structures have been involved in the process of disorganization, and to such an extent as to preclude the possibility of determining to the affection of what structure any particular symptom could be referred. I have been obliged, therefore, to confine myself to a classification which I have thought useful for practical purposes, with a view to the different treatment which the patient may require."

We gave, in a former number, a pretty copious account of the first edition of this work. We shall not go over the same ground again. We shall merely allude to a few of the points introduced for the first time.

1. *Sciatica.*

We have a chapter upon this. We shall take a hint or two.

Three affections, says Mr. Coulson, each distinguished by particular symptoms, have been classified under the term sciatica. In the first the pain is confined to the hip-joint, and all the attendant circumstances clearly demonstrate that the complaint is purely rheumatic. The severity of the symptoms varies with the changes of the weather; when the patient begins to use the limb, the pain is intense, but it partially abates as the exertion is continued, and the heat and circulation of the parts affected are increased and stimulated by exercise. There is not unfrequently rheumatism in some other parts of the body; and if we trace the disease to its origin, we shall find that its exciting cause was such as commonly gives rise to rheumatic affections in general.

With Cotunnius, Mr. Coulson divides sciatica proper into two species, according to the sciatic or crural nerves being the principal seat of the complaint.

The first is characterised by a fixed pain in the hip, chiefly behind the great trochanter, which extends upwards to the os-sacrum, and downwards on the outside of the thigh to the knee. The pain seldom stops at the knee, but often runs on the outer part of the head of the fibula, and descends to the fore part of the leg, where it pursues its course, along the outside of the anterior spine of the tibia, in front of the outer ankle to the dorsum of the foot.

The second species of sciatica, on the other hand, is distinguished by a fixed pain in the groin, which runs along the inside of the thigh and leg, following, in fact, the track of the principal ramifications of the anterior crural nerve, as the other does those of the sciatic.

M. Guerin gives an account of a dissection of inflamed sciatic and scaphenus nerves. The patient died of pneumonia. At the post-mortem examination the right sciatic nerve, from the lower fourth of the thigh, the tibial nerve, to the point where it passes between the gastrocnemii muscles, and the external saphenus nerve, in nearly its whole course, were inflamed.

The inflammation was characterised by a slight redness with serous infiltration, and a moderate degree of tumefaction of the above nerves, particularly of the saphena at its commencement. This nerve was at least double its natural size, of an uniform scarlet colour, and of a hard fleshy texture. In endeavouring to dissect the numerous fibres, both from above and below, towards this spot, they broke, and appeared to be involved in a spongy cord, which was infiltrated with blood, and resistent to the touch; a section of this cord showed nothing but small coagula of blood. In contact with the inflamed saphena nerve, below the gastrocnemii, was a collection of pus, rather effused into the cellular membrane, than enclosed within an abscess, and not penetrating the substance of the nerve.

The filaments of the sciatic and tibial nerves were separated, and as it were dissected, by means of infiltrated serum, to a considerable distance, both above and below the seat of the inflammation.

On the matter of treatment we see nothing to arrest us.

### 2. *Paralytic Affections of the Lower Extremities.*

A mother, says Mr. Coulson, brings her child to a surgeon with supposed disease of the hip: on inquiry he learns that the patient could not walk at the usual period; but that, when eighteen or twenty months old, or even at an earlier age, he was unable to stand, and that the child was at this time cutting the teeth. On examination of the limb, we find it wasted and apparently longer than the other, the nates of the affected side flat, and the temperature of the whole limb below the natural standard. When the child attempts to walk, it cannot raise the limb from the ground, but draws it along; and when it stands, the weight of the whole body is rested on the sound one, while that of the affected side is half bent.

The diagnostic mark of the disease is the absence of any pain in the joint. If we place the child on a table, and press in the neighbourhood of the articulation, or rotate the head of the femur, no pain is produced; whereas, in the disease of the hip, pain would be experienced.

After the period of dentition, the general health is little affected by this complaint; some years, however, commonly elapse before the child recovers much use of the limb.

The effects of this attack are not merely the partial paralysis of the limb, but sometimes considerable distortion. This for the most part makes its appearance in the displacement of the foot from its natural position: certain muscles in particular are affected with paralysis; and the consequence is that the stronger set naturally prevailing, drag the foot in their own direction. If the paralysis affect principally the extensor set of muscles, the heel is drawn upwards, and the foot becomes clubbed; if, on the contrary, the flexors be paralysed, then the extensors prevail, and the foot may be either inverted or everted. The deformity in these cases frequently admits of being relieved by the division of the contracted tendons.

The means of treatment in this complaint consist in endeavouring to restore vigour to the motive powers. The atrophied limb should be assiduously rubbed two or three times a-day; and the patient (if sufficiently old) should be made to exercise the muscles in maintaining and varying the vertical position. This sort of graduated exercise will slowly re-establish a certain degree of power of the weakened muscles, and eventually improve the tone of the entire system. In addition, the affected limb may be immersed in hot salt water for ten minutes daily, and active friction afterwards employed.

Great benefit will also be derived from medical treatment regulated according to the constitution of the patient. In some instances, leeches to the head or spine and brisk purgatives may be required, in others, on the contrary, steel wine, quinine and other tonics, and electricity, will be necessary; but in all cases great perseverance must be exercised, both by the patient and medical attendant, as a long time elapses before any material improvement takes place, even under the most favourable circumstances. But though some benefit may be obtained, a perfect restoration of power and flesh to the wasted muscles can hardly ever be obtained.

"A paralytic affection of one or both of the lower extremities not unfrequently follows chorea when it has been of long standing. Cases of this kind are incurable, particularly if the intellect partakes of the general imbecility; but where the mind remains unimpaired, much may be expected from the adoption of judicious treatment. In addition to the treatment pointed out for the relief of sciatic paralysis, counter-irritants of a more powerful nature are to be employed; and they are more beneficial if applied on the tract of the spinal marrow. It may likewise be observed that chalybeate tonics are more indicated in this case than the preceding; and guaiacum is not so often useful. The bowels in both kinds of the complaint should be duly attended to; but in the latter very active purgatives are from time to time required.

A form of paralysis frequently accompanies hysteria, and that peculiar affection of the hip which has been deemed to be of a hysterical nature. Sir B. Brodie, in speaking of hysterical paralysis, says, that it has this peculiarity: it is not the muscles, in his opinion, that are incapable of obeying the act of volition, but it is that the function of volition is not exercised. How far this theory is well founded is a subject foreign to my present purpose to enter upon; however, the fact cannot be doubted, confirmed as it is by so many analogous examples; viz. that a great extent of muscular inability usually succeeds the cessation of the pain in the hip in this affection."

3. *Congenital Displacement of the Hip-joint.*

Mr. Coulson gives the following summary of the signs which distinguish this from disease of the hip-joint.

1. In congenital lameness, the thigh is, from the first, shortened; in disease of the hip, on the contrary, in the first stage, no alteration from the natural length is perceptible; and afterwards the diseased limb is considerably lengthened before it becomes shorter. The shortening of the limb, also, which is observable in the last stage, is much more considerable than in congenital dislocation.

2. In this congenital malformation, the shortened limb (if the child be put in a horizontal position, and the pelvis fixed with the hand) can, by gentle pulling, be lengthened without any pain; and it immediately becomes short as soon as the extension ceases; in disease of the hip, this is not the case; and the shortened limb cannot be extended without the greatest pain.

3. In the congenital dislocation, the nates of the affected side are either in their natural state or somewhat flatter than in the natural state; on the contrary, when the limb is lengthened, the nates are flat; but when the limb is short, they are tense and projecting.

4. In this malformation the shortened limb, with very few exceptions, is not at the same time thinner and wasted, as is invariably the case in disease of the hip.

5. The motion of the hip is, in congenital dislocation, as free as in the healthy state; the child, with the exception of the lameness, being well and free from pain; in disease of the hip, on the contrary, when the limb is once shortened, the motions of the limb are for ever impaired; the patient occasionally suffers from attacks of fever; and the disease, at least at these periods, is connected with paroxysms of pain.

6. In congenital dislocation, the child, when standing or walking, places the whole surface of the sole of the foot on the ground; those labouring under the disease of the hip rest only on the toes of the affected limb.

Mr. Coulson gives a very full account of the puerperal affections of the joints. We shall merely cite his opinion on their nature.

"The contamination of the blood, I must confess, appears to me the most probable explanation of the varied phenomena of these affections; they really seem, as has been recently expressed, to be the result of a poison 'not confined to certain structures, as the peritoneum or uterus, where its violence is pent up and exhausted, but diffused by the circulation over many organs, causing each to re-act after its own laws, and giving to the disease it produces a character of inextricable confusion and almost hopeless fatality.'"

And though, at one time, we were of a different opinion, we now think so too. We consider this a *very* much improved edition, and can recommend it strongly.

---

Observations on the Surgical Practice of Paris. Illustrated by Cases. Being a Thesis, to which a Gold Medal was assigned by the Senatus Academicus of the Edinburgh University at the Graduation of 1840. By *W. O. Markham*, M.D. S. Highley, London. 1840.

After all the intercourse between this country and the Continent, and after all the journalism of the last half-century, we still know very little of our neighbours, as of ourselves, and whatever improves our acquaintance with them is both instructive and amusing. But this we seem to have learnt—that practical utility is not the god of their idolatry, and that in medicine, or surgery, as in the arts of life, we are in *practice*, their superiors. Their merits are very great, but they are not of this description, and while we cannot but admire the ingenuity, the versatility, and frequent originality of the Frenchman, and the profound abstractions of the German, we must not be misled, however some may endeavour to mislead us, into disparagement and disregard of our own sterling attainments.

Dr. Markham observes:—

"The idea of making any comparison between the merits of the surgery of Paris and of England, never entered the writer's thoughts; and, he confesses freely, that a just and comprehensive decision of this question cannot, with propriety, be drawn from such an imperfect sketch as the following. He is led to make this observation, from remarks which he has already heard fall from those who have perused the manuscript. An attempt to decide such a question cannot but be a task of difficult accomplishment, and one much beyond the writer's powers, and for the obvious reasons, that a lengthened residence in Paris, a discriminating observation, and an impartial and long practised eye, are absolutely necessary for the purpose, qualifications to which he cannot lay claim. Perhaps it might be lawful to doubt if the question ever could be fairly decided by an Englishman. There must be a brighter side in every view; and it is not impossible that the writer may have been, in some instances, only busying himself with the more sombre tints of the picture, or may have made even these darker than true justice demanded, and, at another time, introduced a shadow, where a light should have been found. In plain English, he begs his readers to remember, always, that this is, at best, but a partial view of the matter, written desultorily without any distinct purport, and during the residence of only a few months in Paris."

But let us take a glance at surgery in Paris. We will do our confréres justice, and quote what is for, as what is against them impartially.

*Erysipelas prevalent in Paris.*

Erysipelas prevails to a great extent in all the hospitals of Paris at all seasons of the year, but at intervals it rages with peculiar severity, attacking every even the slightest wound. The situation of an hospital seems not to afford sufficient cause for its occurrence; for during this winter (1839-40) it appeared in several at the same time, and indeed for many weeks bore the character of an epidemic in the different hospitals; and not only in the hospitals, for it was observed by M. Blandin, that at the same moment, and in an equal manner, it was attacking his private patients, subjects of operation. It is impossible to assign as a cause the great heat which is always maintained in the wards here, and the very faulty ventilation—for these circumstances always equally prevail; whether the season of the year may produce any effect, is not clear, but it was prevailing most particularly last year in the Edinburgh Infirmary, during the same months that it was most violent in Paris during the present year 1840, namely, in February and March.

During these months, in M. Blandin's wards, at the Hôtel Dieu, an attack of erysipelas followed every operation, even the simple puncture of a lancet: and, as no operation was ever delayed through fear of its occurrence, the observation was the more evident.

This will be found to be the case, more or less, in all large towns. Weather, no doubt, exercises a great influence—more occult states of atmosphere probably do so too.

*M. Blandin's Crotchets about Erysipelas, and Sangrado Treatment.*

" I said before that M. Blandin never delayed any operation in consequence of the greater prevalence than usual of erysipelas in his wards; and this circumstance must be referred, I apprehend, in some degree to the great confidence which this gentleman has in his mode of combating the disease. He considers erysipelas as an inflammation consisting of two elements—inflammation of the lymphatics, and inflammation of the skin; and avers, that the former invariably precedes the latter, and that, when the inflammation of the skin is apparent, the disease has already made much progress. Upon these ideas his treatment is entirely based; and when the constitutional symptoms, as rigors, vomitings, nausea, &c., indicate an attack of erysipelas, M. B. invariably prescribes the application of leeches in great number, in the neighbourhood or course of the lymphatics, *between* the wound, or part affected, and the trunk: thus, in an injury of the foot, leg, or thigh, the leeches are applied to the groin—of the arm, to the axilla—in threatened erysipelas of the head, to the region of the cervical glands—in a wound of the chest, or, after excision of the mamma, to the axillary and cervical glands—in wounds near the anus, to the groin. Of the efficacy and propriety of this treatment, a great many cases, which I witnessed, seemed to bear evidence. But it also appeared to me, that the very high faith which M. B. reposes in it makes him carry it too far, and use it too exclusively: it seemed sometimes difficult to say, if extensive sloughings, gangrene, and even death of the patient, were not as much to be attributed to the continued application of leeches, loss of blood, and consequent enfeebling of his system, as to the intimate nature of the disease itself; and it is not difficult to conceive, how prejudicial must be this treatment in old, worn constitutions, in individuals enfeebled by disease, or whose powers are prostrated by loss of blood, consequent on operations. Another objection to this treatment is, that it often induces the very ill it is meant to combat: thus, in a case of erysipelas of the hand and wrist, arising after extraction of the metacarpal bone of the thumb, I have seen violent inflammation follow the application of leeches, along the course of the absorbents in the arm, and axilla, and this inflammation has spread to the trunk, and produced death,—at the same time every trace of erysipelas disappearing from the hand and wrist.

I could not feel convinced, also, that inflammation of the lymphatics precedes always that of the skin, for I did not see it demonstrated clearly. This treatment M. B. insists upon most particularly; but I cannot say that it was more successful than many other methods employed, as anointing the part, by M. Velpeau, &c."

*We*, in London, need not say how baseless M. Blandin's theory is—how outrageous his treatment.

*Carelessness with respect to Sponges.*

Any one who has been accustomed to observe the strictness maintained in most English hospitals, to prevent the possibility of any contagious matter being conveyed from one patient to another, through the means of sponges, &c., would be not a little surprised to find the utter disregard which prevails here (at Paris) as to this circumstance: the same sponge which cleans a bubo or a chancre at one bed is carelessly rinsed, and then employed to dress a stump at the next, and in the same manner makes the circuit of the ward. I could never see that sponge squeezed upon a wound, without thinking of the opening of Pandora's box; and I cannot but feel persuaded that I have seen many inflammations, and their severe consequences, which have had at least a very probable origin in this promiscuous intercourse of the sponge.

*How hard it is to operate for Hydrocele.*

"Although the operation for the radical cure of hydrocele may be considered as one of the simplest operations in surgery, there is not one perhaps in which more accidents have happened during its performance; and I should imagine from what I have seen and read, that there are very few surgeons in large practice who have not sometimes in their life made some faux-pas in this operation. The cause of these mishaps it has several times appeared to me, might perhaps be sought in the very simplicity of the operation itself, which may induce the operator to be somewhat less regardful than ordinary in its performance. A collection of the various accidents which have happened in the hands of different individuals in the performance of this operation would form a curious history.

So frequently have these accidents occurred in the practice of some of the Parisian surgeons, that I have heard M. Ricord state, that he believes a kind of atmosphere surrounds the surgeon; a fatality always attends him during the performance of the operation for hydrocele, and that so impressed was Boyer with the truth of this, that he refused to perform it during the latter times of his practice. The principal accidents which happen during the operation are, pushing the trochar into the testicle, injecting into the cellular tissue of the scrotum instead of into the tunica vaginalis, and injecting into the tunica vaginalis when the abdominal rings are not closed.

I have seen M. Blandin do the first; thrust the trochar into the testicle in a small hydrocele, and where, by the aid of a light, the testicle could be distinctly seen lying at the back of the scrotum; on withdrawing the trochar, no liquid escaped, and examination, with a light, showed the trochar sticking in the testicle—of course no injection was thrown in—a violent inflammation, however, followed, abscesses formed, and incisions were made into the scrotum to give issue to the matter; the patient, however, after some weeks perfectly recovered. M. Ricord observed that once, in a great hurry, he sent the trochar right through the testicle of a barber; he saw his error, and reflecting for a moment on the frequency with which these mischances on the testicle terminated without bad results, he, after evacuating the fluid, did not hesitate to inject the tunica vaginalis, the trochar still perforating the testicle; and luckily the barber was cured, without a bad symptom. I should think a prudent surgeon would have equally condemned M. Ricord's hurried practice in the first stage of his operation, as his proceeding in the second; he observed that he had often seen the testicle touched, and not a bad consequence of any kind result. Perhaps the most common error is throwing the injection into the cellular tissue, and this error does not seem to be merely the consequence of the canula having slipped out of the tunica vaginalis, for I believe that I have seen the water of a hydrocele drawn off without the *canula* having entered the tunica vaginalis, which accident might happen either through the maladroitness of the operator, or by reason of the canula not fitting accurately to the trochar. It is not at all difficult to understand how the fluid may escape *from* the hydrocele, when the trochar has *alone* entered the distended sac, and the canula never penetrated it, and how almost impossible it is for the injection to find its way into the contracted sac, or into any part but the cellular tissue. It is clear that this mistake may happen without the surgeon having for a moment permitted the slightest movement of the canula in an outward direction, and may be the cause of no little surprise to the operator, who feels convinced that the canula has never quitted the sac—which, in fact, it has never entered. One of the most curious points in the history of this misadventure is, the very different degrees in which the injury is resented. The natural conclusion of the consequence of an irritating injection thrown into the scrotum, is violent inflammation, and its destructive sequelæ; but, most happily, theory is not always verified by practice in this instance. I have seen in England four ounces of port wine and water injected into the scrotum of an old man of sixty years, and not above a dozen drops return through the canula, and yet the man recovered, without a single bad symptom. M. Ricord threw the injection he generally uses (a solution of iodine) into the scrotum of a young man at the Hôpital du Midi: not one drop ever returned, nor could the most minute examination discover where it was lodged: the expectant treatment was adopted, and, to the utter amazement of all, not the slightest bad result ensued. M. Ricord said, that it was not the first time, by many, that he had seen the same mistake, and the same happy consequence; and mentioned the circumstance rather as a good joke, than as a warning to his auditors."

It is painful to use harsh expressions, but we ask any candid English surgeon whether this is right or decent? Surely the recklessness with which mistakes are incurred, and the sang-froid with which their results are spoken of, evince a disregard of human suffering and human life that ought to meet with the severest reprehension. It is bad in every point of view—bad because it evinces callousness of feeling—bad because it generates indifference to consequences—and while it lowers the moral tone of the professional mind, it interferes with the success and consequently with the real objects of medical science. One reflection must be uppermost in the thoughts of all men of judgement and humanity. Is this the school where our youth are to be trained—is this the source whence English practitioners are to be instructed? We are often invited to turn to the Continent—to yearn after the rich stores of medical knowledge locked up in foreign tongues—to doff our native modes of thought and practice and assume the newer ones of strangers. But those who have paid most attention to the continental schools, have lately drawn less flattering pictures, and couched us of our admiration of the flashy, but the flimsy accomplishments they boast of.

*Are French Records of Cures to be depended on?*

Our author touches lightly but significantly, on this subject, yet the suspicion he gives utterance to does not originate with himself. It is one that has been widely entertained and not unfrequently expressed.

"One cannot suppose, however, that, since accidents of this nature are anything but rare, all terminate thus favourably. Unluckily the annals of medical science contain much oftener the victories than the reverses of its cultivators; for self-love too strongly puts a seal upon the latter; and I do not know if the French medical records are not peculiarly obnoxious to the reproach of this remark; at all events, the analysis of their practice does not always give results consonant with the conclusions they deduce from it."

*Injection of Congenital Hydrocele.*

"One can hardly imagine, how such an error could be made, as injecting a hydrocele, while the mouth of the sac is unclosed—but yet it does sometimes occur. M. Ricord once injected a quantity of wine into the peritoneum, without a shade of a bad result, and he mentioned, that he remembered M. Richerand having done the same thing. Dupuytren says, that this accident has frequently happened,—in one case, induced peritonitis and death: he does not say whether in his own practice or not. I have seen M. Roux inject, in a healthy subject, wine into a sac communicating with the peritoneum, firm pressure being made by three assistants on the canal, and no accident follow. I should observe, that M. Roux did not discover his error of diagnosis, until he had commenced the operation, and was apprized of it by the great quantity of fluid which passed by the canula; and I know not for what reason he afterwards deemed it a case where the sac continued high up into the abdomen, and not communicating with the peritoneal cavity: that it did communicate, the abundance of fluid which escaped seemed to me an evident proof."

M. Ricord appears to commit a very respectable number of blunders, and those not at all little ones.

*Time-keeping Surgery at the Hôtel Dieu.*

The injecting of hydroceles, as performed by MM. Roux and Blandin of the Hôtel Dieu, does not seem to me to be at all guided by scientific principles; or rather, the operation, as to its data, is perfectly incomprehensible, and is only to be accounted for by custom, which in Dupuytren's time always commanded, that the injections for the radical cure of a hydrocele should be three in number at intervals of three minutes each. The above gentlemen practise two injections of warm wine. Modifying the above usage, M. Roux also times the interval in every operation between each injection: the age of the patient, his disposition, his sensation of pain (and the difference of *this* in different individuals is most remarkable), are totally disregarded by him, and the operation is conducted on strictly horological principles. One may be permitted to express surprise that such self-evident errors should be every day repeated before so many observers. If the weak French wine is not irritating enough to produce sufficient inflammation when only once injected, one would have thought that the simple conclusion would have been to seek a more irritating injection, in order that one injection might suffice—that the operation might be simplified and expedited—and, above all, that the double danger of throwing the injection into the cellular tissue might be avoided. Surely, the surgeon who had withdrawn one injection safely, should be too happy at the event. The timing of the process can be no less erroneous. In short, it is clear, that the prejudices of the schools are not altogether destroyed in the performance of this operation.

### *Barbarous Operation for Anchylosis.*

We really, though far from squeamish, sicken while we record the following. A woman, 45 years of age, under M. Blandin's charge at the Hôtel Dieu, had been affected with anchylosis of the knee for ten years, an anchylosis consequent on white swelling. The leg was very much flexed upon the thigh, forming an acute angle with it; motion was almost entirely destroyed; but on rubbing the parts together forcibly, a slight crackling was heard and felt. The back of the condyles, and *not* the lower surface of the femur, rested on the tibia, and the patella was forced on the under surface of the femur, and appeared fixed and united there. All the parts which surrounded, and entered into the composition of the joint, were retracted. Such was the condition of this woman's knee when she presented herself for relief. Her health was good, and she was willing to undergo any suffering, even amputation of the limb, rather than submit to the endurance of this impediment, which rendered her life (always hitherto an active and industrious one) burdensome.

It was evident, that all the simple and ordinary methods employed for the resolution of anchyloses, were futile in respect to this case, both by reason of the long period of the existence of the malady, and its extent. So grave an operation as amputation, M. Blandin thought was inadmissible, though I believe in like cases it has occasionally been performed at the request of the patient.

After some discussion, it was at last determined, at the particular request of the patient, and after many pressing instances from M. Louvrier, that this gentleman should be permitted to practise his operation (which was much the subject of discussion in Paris at the time) on the knee, though still much against M. Blandin's opinion.

The object of M. Louvrier's operation is, by the aid of powerful machinery, to extend the anchylosed joint, disregarding utterly all impediments, and the nature of these impediments. The apparatus employed, it is impossible to describe, as it is of infinite complexity, and requires much time, labour, and dexterity to arrange; but the principle of its action is plain: The thigh is made a fixed point, and extension is applied to the leg by aid of the two mechanical powers, the screw and the lever, sufficient to reduce the limb from its highly flexed, to a perfectly extended position. What is going on in or about the joint, during the operation, it is impossible to observe, as the whole limb is thickly enveloped in coverings of brass, leather, &c. &c. When the apparatus is adjusted, all that is visible is the limb thus covered, placed in a wooden case, and resting in a kind of groove, where it slides as it is extended. The operator places himself at the foot of the apparatus, and turns a small wheel, which acts on the leg through the medium of a very strong catgut cord attached to different parts of the leg; the limb gradually redresses itself, and in about two minutes it is perfectly extended. The pain suffered by the patient seemed excessive, and was prolonged by reason of the apparatus breaking in some part during the first attempt. What the force applied was I do not know, but it must have been very great. On examining the limb, when the apparatus was removed, the patella was found free, and the tibia almost entirely thrown *behind* the femur. Conjecture alone could give information as to what had taken place inside the joint. The skin was not torn near the joint, but it was at the heel. Every precaution, after the operation, was taken to anticipate the violent inflammation which might be expected to arise, by the application of leeches, &c., and by placing the limb in a perfectly immoveable position. By these, and other antiphlogistic means, the violent constitutional symptoms that arose were subdued, and she (the patient) seemed to rally from the low state into which she had fallen. But it was only an appearance of return to health, and she gradually sunk into a desponding state. The pain in the knee was constant, her nights were often sleepless, her appetite not good, pulse quick, and extreme pallor of the face. She thus continued till she left the hospital, about five months afterwards. The limb then was perfectly useless, and always painful, and could not sustain the slightest pressure of the body; and this woman evidently seemed sinking from some cause, when she demanded her dismissal. It was difficult to give the precise state of the knee, as it was, and had been all along from the time of the operation, enveloped in the starched bandage.

Two other operations of this nature were performed shortly afterwards, on patients of M. Velpeau at La Charité; one died fifteen days after the operation, it was said, of peritonitis; the other, after a rather longer period. At Hôpital Neckar, another case terminated fatally, through gangrene, I believe, of the limb; and M. Roux mentioned a case where he was called in to perform amputation of the thigh, which had been fractured in a young man during M. Louvrier's operation.

Several other cases were mentioned in the journals as occurring in hospital and private practice, and with different success as to their results.

Comment on such ignorance and barbarity is superfluous.

---

### MR. HAWES'S MEDICAL REFORM BILL.

The Bill before us came before the public after the latter portion of our Journal closed. We are under the necessity of introducing it here and displacing, on account of it, some Notices of New Works. The Bill having a present application and interest, this will not, perhaps, be regretted.

A Bill to amend the Laws relating to the Medical Profession in Great Britain and Ireland.

1. *Preamble.*—Whereas it would tend to the advantage of the public to alter and amend *the laws* touching the medical profession, and to make due provision for the *prevention* of persons, not being duly qualified, from practising the art of medicine, and to provide that *all persons before practising the same* shall be duly *examined,* as to their skill and knowledge, by learned and competent persons, and *thereupon* be permitted to follow and exercise the art of medicine in any part of the British dominions.

2. *Interpretations.*—Be it therefore enacted, That in this Act the words, "art of medicine" include within their meaning the recommending, prescribing, or ordering, either directly or indirectly, any *medicine, remedy,* or *application,* whatsoever, for the relief or cure of any disorder, ailment, or illness of the body or mind, or any part thereof; or performing any *surgical operation,* minor or capital; or practising *midwifery;* and the words "medical practitioner" mean a person *qualified under this Act* to practise the art of medicine; and that the words "chemist and druggist" mean a person who shall sell, deal in, mix, or dispense for sale, any drug or medicine for the cure or relief of any bodily disorder, ailment, or illness, except such person as shall have obtained a certificate to practise the art of medicine; and the word "England" shall include Wales.

3. *The Registrars of Medical Practitioners.*—And be it enacted, That the Secretary of State shall, within three months from the passing of this Act, appoint three persons to be registrars, one for England, to have his office in London; another for Scotland, to have his office in Edinburgh; and the third for Ireland, to have his office in Dublin; and also such clerks as the said secretary shall deem necessary for carrying this Act into execution, until the election of the first councils hereinafter provided; the said Secretary of State may also remove any person so appointed. Before the election of the said councils all expenses for carrying this Act into execution shall be paid by the lords of the Treasury out of any monies received by the said registrars by virtue of this Act.

4. *Persons already qualified to Practise Medicine.*—And be it enacted, That the registrars shall, within thirty days after their appointment, distribute certificates to practise medicine, according to the form annexed, to every person who shall apply for the same, and who *shall produce his diploma,* certificate, or licence to practise medicine or surgery, dated prior to the passing of this Act, granted by any English, Scotch, or Irish university, college, hall, or other person or corporation legally entitled to grant the same at the time of the passing of this Act, and also to every person who shall apply for the same, and who was actually practising medicine in England prior to August, 1815; but the said registrars shall grant such certificate for that part of the kingdom only for which they shall severally be appointed to act.

I authorise [Thomas Johnstone] of [15, Cavendish-square], to practise the art of medicine in England (or Scotland or Ireland) until February 1st, 1850.

5. *Expense of Certificates and Registers of Practitioners.*—And be it enacted, That every person applying for a certificate to practise the art of medicine shall pay for it to the registrar the sum of [ ], and be subject to the like payment annually. The certificate shall bear date on the day on which it is granted, and continue in force until Feb. 1st, 1843; and the said registrars shall, at any time during one month preceding the 1st of February in every year, grant such certificates, and also to such other persons as shall be entitled to them, to take effect from the date thereof, and continuing in force until the 1st of February in the year next following that in which they are granted; and each registrar shall register in a book every certificate which he grants, and in February of every year shall print a list of all persons to whom he has granted such certificates prior to the 1st of February for the year ensuing, and of their places of abode; and the persons only whose names are contained in the last printed medical lists shall vote at any election held for the purposes of this Act. Copies of such medical lists shall be furnished to any person for sixpence each copy.

6. *Councils, and who shall elect them.*—And be it enacted, That on the 1st of June, 1842, and once in every three years, the medical practitioners registered and resident in England, shall elect a council for England; those in Scotland shall elect a council for Scotland; and those in Ireland shall elect a council for Ireland; twenty councillors for each council, who, in three whole years, shall go out of office, each councillor being capable of being re-elected.

7. *Who shall recommend the Councillors, and how.*—And be it enacted, That forty days previous to each election any such voter may transmit to the registrar a paper, as annexed, signed by six or more voters, containing the names of any persons he may wish to be elected as councillors, and the registrar shall print all such names together (unless they be members then going out of office) with the names of the persons recommending him, and only persons so nominated shall be eligible to be elected.

8. *Places of Elections.*—The elections shall be held at such places as the respective councils may appoint, within four miles of the General Post-office in London, within two miles of that office in Edinburgh, and within two miles of the same in Dublin; and the registrars shall give public notice of the times and places in the newspapers ten days before the elections. The *first* election shall be held at such places as the registrars shall appoint.

9. *Place, Time, and Mode of Voting.*—The elections shall be held before the registrar, commencing at 9 o'clock, A.M., and closing at 4 o'clock, P.M. of the same day, and every voter may vote for the whole number of councillors to be chosen, by delivering to the registrar or his deputy a voting paper according to the form annexed, in the Schedule, folded or sealed up, so that the contents cannot be seen, containing the names of the persons for whom he votes; or if sent by post, then the said voting paper shall be inclosed in a declaration, according to the form annexed; and the registrars shall transmit to every voter, fourteen days before the election, one voting paper and declaration to be returned, filled up, to the registrar on the day of election, to be opened according to the provisions hereinafter made.

10. *Who shall conduct the Elections.*—After the said election the registrar shall proceed to examine the voting papers to ascertain who are elected, and the twenty persons who have the greatest number of votes shall be the councillors elected; in case of an equality in the number of votes for any two or more persons, the respective registrars shall publicly draw by lot from among those having an equality of votes, as many names as may be necessary to complete the whole number of councillors to be elected; and the names of the council shall be published in the London, Edinburgh, or Dublin newspapers having the largest circulation, in seven days after such election; and each of the past councils shall, after the year 1842, appoint two or more councillors, to see and certify that the said election has been fairly conducted. At the elections for councils in 1842, the Secretary of State shall appoint two persons to superintend the election.

11. *Existing Medical Colleges and Corporations,*—And be it enacted, that the University of Oxford, in convocation assembled, and the Senates of the Universities of Cambridge and London, and the *Fellows* of the London College of Physicians, and the *Council* of the London College of Surgeons, and the Court of Assistants of the Apothecaries' Society in London, shall, if they think fit, severally appoint one fit person to be a member of the council for England; and the Faculties of Medicine in the Universities of Edinburgh, Glasgow, St. Andrew's, and Aberdeen, and the Councils of the Edinburgh Colleges of Physicians and Surgeons, and of the Glasgow Faculty of Physicians and Surgeons, shall, if they think fit, severally appoint one fit person to be a member of the council for Scotland; and the Provost and Fellows of Trinity College, Dublin, and the Councils of the King's and Queen's College of Physicians in Ireland, and the Dublin College of Surgeons, and the Dublin Court of Apothecaries' Hall, shall, if they think fit, severally appoint one fit person to be a member of the council for Ireland.

12. *Future Registrars.*—Upon any vacancy in the office of registrar the several councils shall appoint another registrar; or, failing so to do, the Secretary of State shall appoint one.

13. *Removal of Registrars—New Registrars—Deputies.*—The council of each kingdom respectively may remove any registrar for neglect, misconduct, or infirmity of body or mind; and in the event of the office being at any time vacant, such vacancy shall be advertised in newspapers of the largest circulation within the kingdom to which he acted, six weeks previous to the day for electing a successor. Candidates shall give notice of their intention to the council fourteen days previous to the election. If any registrar is absent, or incapable for a time, the president of the council shall appoint a good deputy to act for him.

14. *Officers under the Act.*—On the 14th of June in every year each of the councils shall elect, by ballot, a president of their council for one year; and if he die, or cease to hold the office, the council shall, within ten days, elect by ballot another president for the remainder of the year; and shall also appoint a treasurer, not being a member of the council; and also three auditors of the accounts of the council; and also their own clerks and other officers. The councils may remove the treasurer, clerk, officer, or servant so appointed, and appoint other persons to fill those offices when vacant; and shall fix the salaries of the registrar, treasurer, and others so employed; and also such remuneration to the individual members of council for the execution of their duties as they shall deem reasonable.

15. *Meetings and voting of Councils.*—All acts of the councils may be decided upon by the majority present, the meeting consisting of not less than a majority of the whole council. The president, if present, shall have a casting vote. The meeting of any council shall be notified three days beforehand, by summons sent to the abode of each member.

16. This clause provides for the holding of *special meetings.*

17. *Monies of the council.*—All monies, fees, and dues belonging to the council, shall be paid to the treasurer, and carried to the account of a fund to be called "the medical fund" and applied towards the payment of all expenses incurred in carrying this act into effect; and in case "the medical fund" shall be more than sufficient for these purposes, the surplus may be applied from time to time towards the encouragement and advancement of medicine, and of science and literature connected therewith, as the senate hereinafter mentioned, and council conjointly, shall think most desirable.

18. *Payments.*—The treasurer shall pay no money out of the fund, save upon the order of three or more members of the council, countersigned by their registrar, except as hereinafter provided.

19. *Accounts.*—The treasurers' and registrars' accounts shall, in June and December in every year, be properly audited and published, along with the current medical list.

20. *The councils when at Law.*—The councils may sue and be sued in the name of their registrar for the time being, who shall be deemed plaintiff or defendant in every such suit or action: provided also, that the registrar, although appearing as plaintiff or defendant on the record, may, if not otherwise interested or objectionable, be a witness in every such action or suit.

21. *Election of a Medical Senate.*—And be it enacted, That each council shall, within thirty days next after their election in 1842, and in the December of every fifth succeeding year, elect by ballot from amongst themselves, or others, three persons, who shall be called "the Medical Senate of Great Britain and Ireland," and continue in office five years. Vacancies in the senate shall be filled up by the councils for the remainder of the five years. Members of senate shall be capable of being re-elected.

22. *Its Registrar.*—The registrar for England shall be the registrar to the senate.

23. *Meetings of the Senate.*—The senate shall, on the 10th of July in every year, meet in London, at the usual place of meeting of the council for England; and all reasonable expenses of the members of the senate attending any such meeting, shall be defrayed out of the "medical fund" of the respective councils. (The rest of the clause provides for the holding of special meetings of the senate at the desire of any two of the councils.)

24.—*A majority* of the whole senate must be present, and assenting to meetings and acts of the senate.

25. *The President.*—At the annual meeting of the senate, it shall elect by ballot its own president for one whole year. A vacancy to be supplied, in the same way, for any portion of the twelve months.

26. *Privileges of Senators.*—Any member of the senate may be present at any meeting of the councils, and take part in its discussions, but not vote there; and also be present at any examination held by any examiners appointed under this Act.

27. *Expenses of the Senate.*—All expenses of the senate (excepting the expenses of its members attending the same as hereinbefore provided) shall be divided into three equal portions; and the senate shall make an order, signed by three of its members and countersigned by their registrar, for the payment of the one such portion upon each of the treasurers of the several councils, who shall pay the sums expressed in such orders out of any monies coming into their hands by virtue of this Act.

28. *By-laws relating to Medical Education and Examinations for Diplomas.*—And be it enacted, That the said senate shall maks such by-laws for the United Kingdom, regulating in all respects the *education* of candidates applying to be examined for a diploma of qualification to practise the art of medicine, or to carry on the trade and business of a chemist and druggist, and also regulating the *examinations* of persons prior to the granting of the said diploma, as from time to time shall to the said senate seem proper; and such persons only as shall comply with such by-laws, shall be admitted to such examinations. But no such by-law shall be of force until forty days after a copy thereof shall have been published in the *London Gazette*, and another copy thereof sent to one of her Majesty's principal Secretaries of State for the time being; and if at any time her Majesty, with the advice of her Majesty's privy council, shall disallow such by-laws, or any part thereof, such by-laws or the part thereof, so disallowed, shall be null and void. If the senate neglect to publish any such by-laws within twelve months, the Secretary of State shall make and publish them: provided also, that any such by-laws shall not be valid unless they require that previous to the examination of any person desirous of obtaining a diploma of qualification to practise the art of medicine, he shall *produce a diploma, certificate, or letters testimonial, of having taken a degree in medicine, or of having passed an examination in medicine or surgery before some univeristy, college, hall, or other persons, legally entitled to grant a diploma, certificate, or letters testimonial, at the time of the passing of the Act.*

29. *New Pharmacopœia.*—And be it enacted, That the senate shall cause their registrar to publish, under their direction and authority, a book containing a list of medicines and compounds, and the manner of preparing them, together with the true weights and measures by which they are to be prepared and mixed, &c. to be called "The British Pharmacopœia;" and that the senate may alter and amend such Pharmacopœia as often as they shall deem necessary; and every chemist and druggist shall mix, make, and compound, according to the directions therein contained, and according to no other formula, and obey in all respects the orders therein directed.

30. *The only future Medical Practitioners, Chemists, and Druggists.*—And be it enacted, That no male person whatsoever shall, from the 1st of February, 1842, practise medicine for remuneration, directly or indirectly, in any part of the United Kingdom unless he has obtained a certificate to practise the said art according to the provisions of this Act; nor shall any person from the 1st of December, 1842, carrying on the business of a chemist and druggist in any part of the United Kingdom unless he has obtained a licence under this Act.

31. *The present Licencing Bodies.*—And be it enacted, That after the publication of the by-laws for regulating the examinations for diplomas to practise medicine, no corporation nor any university, except under this Act, shall grant any diploma, certificate, or licence to practise medicine, or to carry on the trade of a chemist or druggist in any part of the United Kingdom of Great Britain and Ireland.

32. *Examiners and Successful Candidates.*—And be it enacted, That each of the councils shall appoint annually fit persons to be examiners for granting diplomas in medicine, or licences to carry on the business of a chemist and druggist; and such examiners shall examine as the senate may direct, all candidates who may prove to be duly qualified according to the by-laws of the senate, and report the result of the said examinations to their respective councils, who shall direct their registrar to grant a diploma to practise medicine, or carry on the trade of chemist and druggist according to the forms annexed, to every person whom the council shall deem qualified, upon the payment of [ ] for such diploma; and every person who shall have obtained such diploma may obtain from one of the registrars a certificate or licence to practise medicine, or trade as a chemist and druggist, and to renew it annually; and no person, except as herein provided, shall receive such a certificate or licence unless he pass such examinations aforesaid.

33. *Navy and Army Surgeons.*—Every person desirous of being appointed medical officer in her Majesty's army or navy shall be eligible for such appointment upon obtaining a diploma of qualification, or a certificate to practise the art of medicine, and, while actually employed on her Majesty's service, shall not be liable to renew it annually.

34. Dentists or cuppers now in practice shall, before the 31st of December, 1842, send a declaration as annexed to that effect, signed by themselves and two substantial householders, to the registrar, and shall obtain a certificate giving them leave to practise still what they practised before.

*Declaration of Dentists and Cuppers.*

I, *A. B.*, of *C.*, in the county of *D.*, hereby declare that I was practising as a (dentist or cupper) in the county of *D.*, previous to the 1st day of [ ]

(Dated and signed).

35. And be it enacted, That each registrar shall grant a licence to carry on the business of chemist and druggist, according to the form annexed, to every person who has received a diploma of qualification from either of the councils, upon the payment of the sum of [ ] for such licence, to be renewed annually, on the 30th day of November; and each registrar shall register every licence which he grants as aforesaid, and publish the same in his medical list; and also the registrar shall grant the same to persons already, before the passing of this Act, carrying on the trade of chemist and druggist, or persons being assistants or apprentices, and applying within twelve months to the registrar for the same, and these persons are not to renew it annually unless they desire: provided always, that if any person as aforesaid shall *once* renew his licence as aforesaid, it shall not be lawful for him to carry on the trade and business of chemist and druggist unless he continue to renew it annually.

*Licence to carry on the Trade and Business of Chemist and Druggist.*

I, *A, B.*, by virtue of the powers vested in me by an Act of Parliament passed in the year of the reign of her Majesty Queen Victoria, authorise *C. D.* of to carry on the trade and business of a chemist and druggist in that part of the United Kingdom of Great Britain and Ireland called until the 31st day of November, 18 .

Dated and signed by the medical registrar.

*Licence to carry on the Trade and Business of a Chemist and Druggist.*

I, *A. B.* by virtue of the power vested in me by an Act of Parliament, passed in the year of the reign of her Majesty Queen Victoria, hereby authorise *C. D.*, of to carry on the trade and business of a chemist and druggist, he having signed a declaration that he was engaged in the said business previous to the passing of the said Act.

Dated and signed by the medical registrar.

*Declaration of Chemists and Druggists.*

To the medical registrar for

I, *A. B.*, of , hereby declare that I was previous to the day of 18 , at . Dated and signed.

36. *Medical and Chemical Assistants and Apprentices.*—And be it enacted, That every medical practitioner, and every chemist and druggist, having any assistant or apprentice in his employ shall, before the 31st of December in every year, transmit to the registrar the name of such assistant or apprentice, according to the form annexed, together with a declaration in writing, signed by himself, according to the said form; and the registrar is required to register in a book the names of the persons so transmitted to him, and insert them in the medical list published next after the receipt of such declarations; and be it enacted, that no medical practitioner shall, after the 31st day of December, 1842, employ any person as an assistant who does not possess a diploma of qualification, or a certificate to practise the art of medicine; and no chemist and druggist shall employ any person to assist him in the actual vending of drugs and medicines who does not possess a diploma of qualification to carry on the trade and business of a chemist and druggist, or a licence according to the form above, unless such persons so being assistants to any medical practitioner or chemist and druggist shall be apprentices for any period not exceeding seven years, and duly registered: provided always, that any person being an assistant to any medical practitioner, or to any chemist and druggist, shall not be required to take out his annual certificate or licence during the time he shall be so actually employed.

*Declaration of Assistants and Apprentices.*

To the medical registrar for

I, *A. B.*, of declare that I have in my employ [Insert christian and surname; age; whether assistant or apprentince; if apprentice, date of indentures; if assistant, date of diploma]; and I request that you will duly register the same. (Signed)

N.B.—The person making such declaration must state whether he is a medical practitioner or a chemist and druggist. This declaration must be sent to the r gistrar before the 31st of November annually.

37. *Power and Privileges of Medical Practitioners.*—And whereas certain powers, benefits, privileges, appointments, acts and things, have, by custom, law, or right, been conferred on, or executed, or performed by physicians, or surgeons, or apothecaries, be it therefore enacted, That from and after the 1st of February, 1842, every power, privilege, appointment, and authority, heretofore held by any physician or surgeon, or apothecary, by any law, charter, or custom, shall be held and enjoyed by persons possessing certificates to practise the art of medicine according to the provisions of this Act, and such persons only.

38. *Money Accounts.*—And whereas disputes and differences have arisen as to the right of medical practitioners to recover at law charges for professional visits and consultations, be it therefore enacted, That any person duly qualified to practise the art of medicine may recover in any court of law by action of promises, or debt, any reasonable sum of money for professional visits and consultations, together with full costs of suit.

39. Medical practitioners, and chemists and druggists, shall be exempt from serving as *jurors* and *other officers*, after the passing of this Act.

40. This clause imposes a penalty of £100 on any registrar or his deputy refusing or neglecting any duty enjoined by this Act.

41. This clause imposes a penalty of twelve month's imprisonment for illegally obtaining diplomas, certificates or licences from any registrar.

42. This clause imposes a penalty of six months' imprisonment for making a false declaration under this Act.

43. This clause imposes a penalty of £20 (each offence) for illegally practising medicine, or carrying on the trade of chemist and druggist.

44. And be it enacted, That every medical practitioner and every chemist and druggist who shall employ any assistant not being duly qualified according to the provisions of this Act, or who shall neglect to make a declaration of any person being an assistant or apprentice in his employ according to the provisions hereinbefore contained; and every person not being duly qualified according to the provisions hereinbefore contained, who shall act as an assistant to any medical practitioner or chemist and druggist, shall pay for every such offence any sum not exceeding ten pounds.

45. This clause states that offences under this Act may be prosecuted before any two of her Majesty's justices of the peace in petty sessions assembled acting in and for the county in which the offence has been committed, &c.

46. And be it enacted, That in every adjudication of a penalty under this Act, and nonpayment thereof, it shall be lawful for the magistrate to commit the offender to any gaol within his jurisdiction, for one calendar month where the sum shall not exceed five pounds, and for three calendar months where the sum shall exceed five pounds, the imprisonment to cease on payment of the sum due, and the costs for the recovery thereof; and the one moiety of every penalty recovered shall be paid to the informer, and the remainder to the treasurer of the medical council for that part of the United Kingdom in which the offence was committed.

47. Any person who may think himself aggrieved by such conviction may appeal to the justices of peace at the next general or quarter sessions of the peace, under such restrictions as are usual or proper in cases of appeal against convictions before magistrates.

48. Business that may happen to fall on a *Sunday* shall be done on the Monday following.

49. And be it enacted, That this Act may be *amended* or *repealed* by any Act to be passed in the present session of Parliament.

